# The primary stability of different implants for intra-articular calcaneal fractures: an in vitro study

**DOI:** 10.1186/s12938-018-0484-6

**Published:** 2018-05-02

**Authors:** Ming Ni, Jiong Mei, Kun Li, Wenxin Niu, Ming Zhang

**Affiliations:** 1 Department of Orthopedics, Pudong New Area People’s Hospital, Shanghai, China; 2 Shanghai Yangzhi Rehabilitation Hospital, Tongji University School of Medicine, No. 2209, Guangxing Rd, Shanghai, 201619 China; 3 Department of Orthopedics, Tongji Hospital, Tongji University School of Medicine, Shanghai, China; 4 Department of Biomedical Engineering, The Hong Kong Polytechnic University, Hong Kong, China

**Keywords:** Calcaneal fractures, Absorbable screw, Fracture fixation, Internal fixation, In-vitro experiment

## Abstract

**Background:**

Calcaneal fractures account for around 2% of all fractures and most of them are intra-articular fractures. Many implants have been used in the fixation of calcaneal fractures, but their biomechanical stability has not yet been well investigated. The aim of this study was to compare the primary stability of four fixations of calcaneal fracture.

**Methods:**

Eight cadaveric calcaneus samples were used to simulate the Sanders’ types III fracture pattern and fixed through four different implants, namely, K-wires, cannulated screws (CS), absorbable screws (AS), and plate-screw system (PSS). Each specimen was then placed into a custom-made jig and was loaded through a material testing machine to simulate the physiological condition. The primary stability was measured in the vertical direction as the stiffness and anterior–posterior direction as the calcaneocuboid force. One-way analysis of variance was used for data analysis.

**Results:**

The results showed the highest stiffness of 634 (383–891; SD 226) N/mm in the intact model. It was significantly higher than the models fixed with K-wires, CS or PSS. There was no significant difference in vertical stiffness between fractures fixed with AS and the intact model or other fixed models. The intact model showed the lowest calcaneocuboid force of 153 (120–218; SD 39) N, while the fractures fixed with AS showed the greatest force of 242 (146–398; SD 84) N. The significance was only detected between these two models.

**Conclusions:**

The global stiffness was similar when the calcaneal fractures were fixed by K-wires, CS and PSS. The stability of the AS fixation differed along both the vertical and anterior–posterior directions, and was greatly influenced by the bone quality. AS for fracture fixation should be designed with greater strength and pull-out resistance.

## Background

Calcaneal fractures account for around 2% of all fractures presented to emergency departments and approximately 75% of these are intra-articular fractures involving the posterior subtalar joint [1]. Treatments for displaced intra-articular calcaneal fractures can be divided into conservative and operative management [2–4]. A review of treatment options showed that patients may have superior outcomes with surgical fixation [5].

The goals of surgical treatment of calcaneal fractures are to reduce fractures and articular congruity, provide stable fixation, and regain motion early in the postoperative period. A number of implants, like the Kirschner wires (K-wires), absorbable screws (AS), cannulated screws (CS), and plate-screw system (PSS), have been successfully used for the internal fixation of calcaneal fractures [6–9]. The stability of the fixation is an important factor in maintaining the position of the reduction, but previous biomechanical studies were mostly concerned about plate selection and implant type [10–14]. The biomechanical stability of different devices has not yet been well investigated and there is no clear evidence which one will produce the best stability for calcaneal fractures. In previous studies, vertical stiffness was the only parameter considered when gauging the biomechanical stability of internal fixation. Obviously, more indexes should be considered when evaluating such multi-axial movements.

The purpose of this study is to compare the biomechanical stability of intra-articular calcaneal fractures after fixation with four common implants: K-wires, AS, CS, and PSS. Fixation was tested in both vertical and horizontal directions. Because of the insufficient stiffness of absorbable material, the null hypothesis was that fixation with K-wires, CS and PSS would be biomechanically superior to fixation with AS.

## Methods

### Preparation of cadaveric specimens

Eight calcaneus specimens were harvested from fresh cadavers. Four right feet and four left feet were used. The donors had a mean age at death of 50.5 (38–72) years and the mean foot length was 24.4 (23.8–25.6) cm. Radiographs were taken of each specimen to rule out bone abnormalities and previous injuries. The specimens were present and stored at − 20 °C and thawed at room temperature for 24 h before testing. The calcanei were cleaned of all soft tissues before simulation and fixation.

#### Fracture model and fixation

The Sanders’ type III calcaneal fractures were simulated as described by Smerek et al. [15]. Firstly, a V-shaped osteotomy was made near the angle of Gissane in the coronal plane which divided the calcaneus into three fragments. The central fragment contained most of the posterior facet. Next, two sagittal osteotomies were made on the central fragment which divided it into three parts: the sustentaculum, and the middle and internal posterior facet.

After the osteotomy, the fractures were fixed using four different implants: K-wires, CS, AS, and PSS. The smooth K-wires (Puwei Ltd., Shanghai, China) had a diameter of 2-mm. The CS (Puwei Ltd., Shanghai, China) were titanium, short-threaded screws, with diameters of 4.0- and 7.3-mm. The AS (Dikang Biomedical Co., Ltd., Chengdu, China) were made from Poly-l-lactide acid (PLLA) and had diameters of 4.0- and 7.3-mm. The PSS (Puwei Ltd., Shanghai, China) was a non-locking stainless steel AO “Sanders plate” with cancellous screws.

The K-wires and screws were inserted in a cross configuration. Two wires were inserted from the lateral side beneath the posterior facet to the sustentaculum tali and two from calcaneal tuberosity in the direction of the calcaneocuboid joint but not penetrated into joint. The plate was fixed using three screws beneath the posterior facet, three in the posterior process and two in the anterior process. All screws were inserted in a bicortical manner. The middle screw under the posterior facet was directed to reach the sustentaculum in all cases.

### Biomechanical tests

**Fig. 1:**
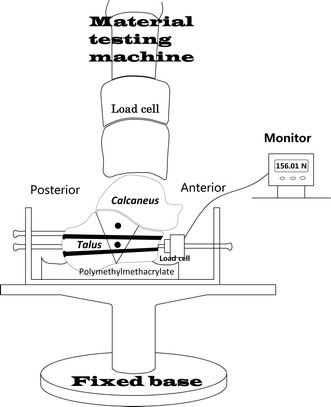
Schematic of the testing equipment. A round polyester resin platform was placed on top of the talus, and the calcaneus was firmly fixed into the jig. This positioning of the calcaneus was maintained with polymethylmethacrylate. The force on the calcaneocuboid joint was measured by a load-cell sensor. A pre-programmed material testing machine controlled the compressive loading

For mechanical testing, the posteroinferior calcaneal tuberosity was potted in a holder using polymethylmethacrylate (Technovit 3040; Heraeus Kulzer GmbH, Wehrheim, Germany). The calcaneus was inclined at 15° and the hindfoot was maintained at 90° to the horizontal plane of the holder to simulate the anatomical position of the heel. Each specimen was then placed into a custom-made jig. The calcaneus was firmly fixed with two metal boards and six locked screws. The anterior board could move in the mediolateral direction and posterior in the anterior–posterior (AP) direction to obtain the appropriate positioning of the calcaneus. As shown in Fig. 1, the calcaneocuboid force was measured by a load-cell sensor (Xinjingcheng, Shenzhen, China) with the range of 0–1000 N. The sensor was placed in the center of the joint and was bolted to the jig.

All tests were performed on a material testing machine (CSS-44000; CRIMS Co., Ltd., Changchun, China). The load was applied and transmitted through the talus which was placed on the upper surface of the calcaneus (Fig. 1). Each specimen was tested under five operating conditions in the following order: (1) intact calcaneus; (2) calcaneal fracture fixed by K-wires; (3) fracture fixed by CS; (4) fracture fixed by AS; and (5) fracture fixed by PSS. When one fixation model was tested, the implants were removed. This order was based on the morphological features and size of four fixation implants in order to avoid the influence of earlier operations. The CS were used prior to AS because some bones could be remained along the hollow threaded rod.

### Data acquisition

The specimens were preconditioned by axial loading (0–200 N) through five cycles at the rate of 2 mm/min before formal testing of each model. Specimens were then subjected to axial stepwise loading. The load–displacement curve of calcaneus was continuously recorded by a transducer. When the load through the material testing machine increased, the calcaneocuboid force would also increase and its value could be read through a monitor (Fig. 1). The maximum value was regcorded as the peak force of calcaneocuboid joint. The calcaneocuboid peak pressure has been found to successfully assess stability of normal foot, flat foot and corrected flatfoot deformity [16]. Because the contact force through this joint represents the AP mechanical effect, it should be used as an index of the stability in this direction. The reproducibility of the loading procedure was tested by sequentially loading the same specimen three consecutive times while maintaining all other parameters constant. The average value of three tests was used for further statistical analysis. Then, each calcaneus bone was tested 15 times.

### Statistical analysis

Statistical analysis was performed using Statistical Package for Social Sciences (SPSS) for Windows, Version 18.0.1 (SPSS Inc., Chicago, IL, USA). One-way analysis of variance (ANOVA) was used for data analysis. When significant differences occurred during the ANOVA test, a post hoc multiple was used to locate the differences between the different specimens. The null hypothesis at the level *p* < 0.05 was that there were differences between the different implants.

## Results

**Fig. 2:**
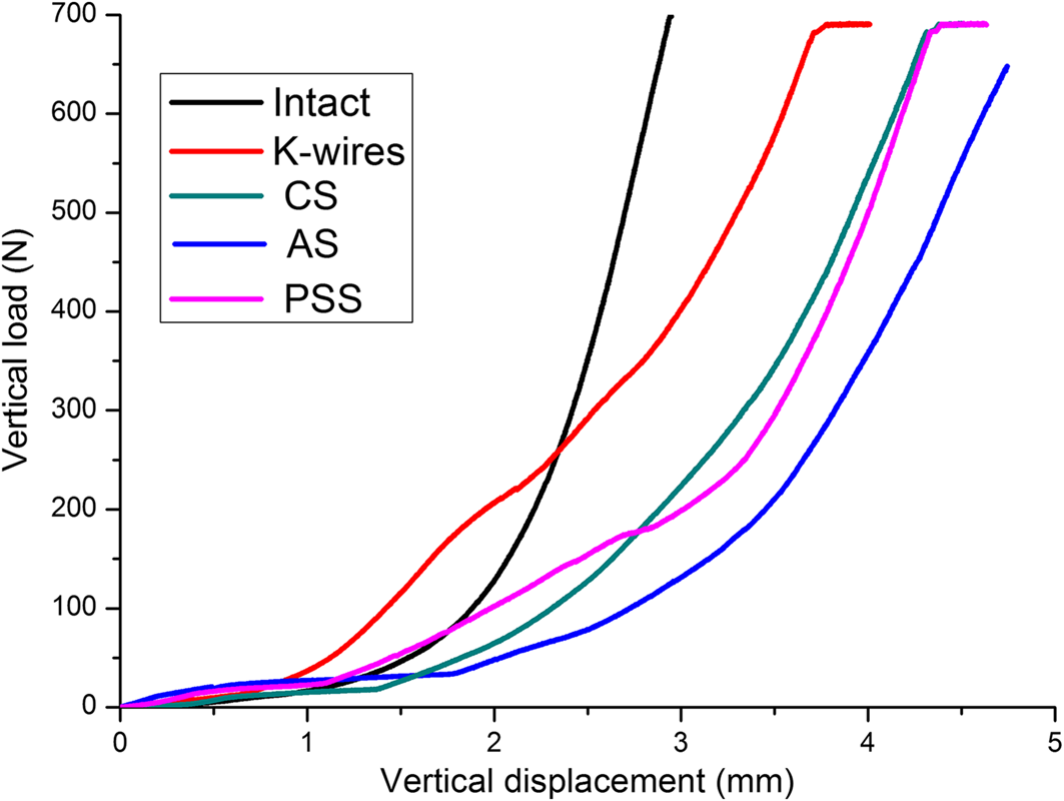
The load–displacement curves of intact and four fixation models for a representative sample. *CS* cannulated screws, *AS* absorbable screws, *PSS* plate-screw system

**Fig. 3:**
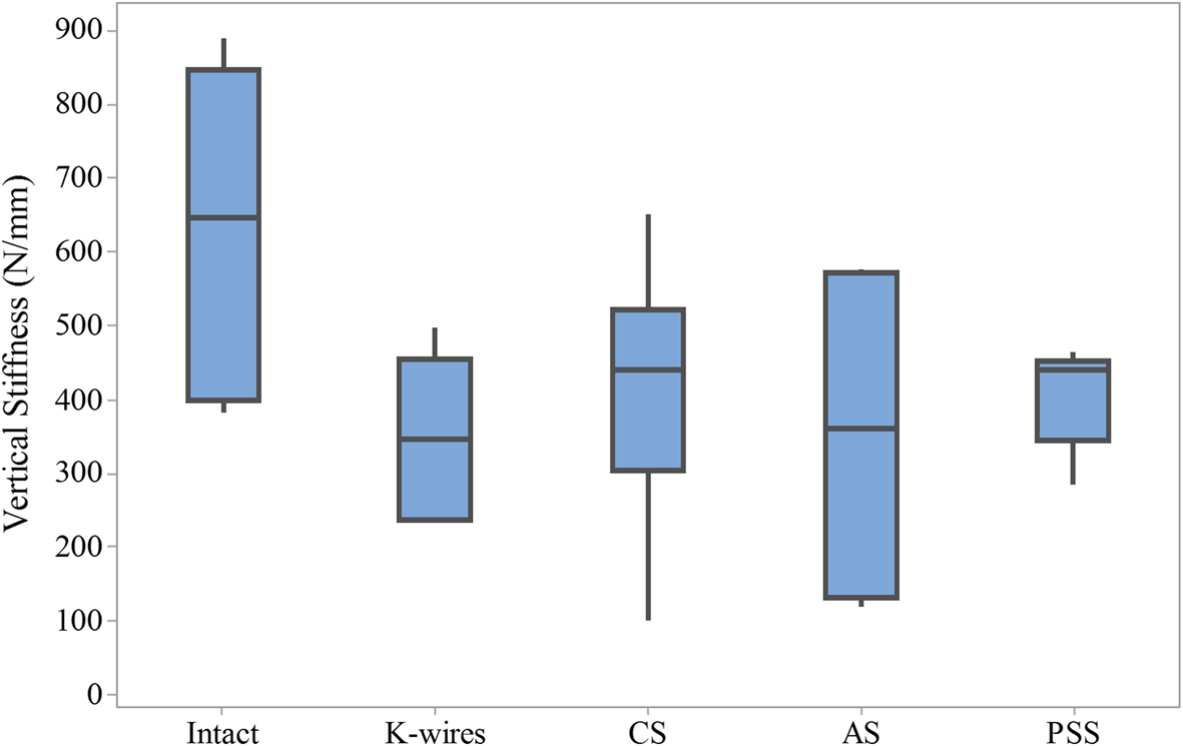
Box and whisker plots for the vertical stiffness of five conditions. The box indicates the interquartile range. *CS* cannulated screws, *AS* absorbable screws, *PSS* plate-screw system

None of calcaneal fracture fixations tested failed during the vertical loading. The load–displacement curves of a representative sample were shown as Fig. 2. A comparison of vertical stiffness among different models is shown in Fig. 3. The intact model showed the highest stiffness of 634 (383–891; SD 226) N/mm. It was significantly higher than the models fixed with K-wires, CS or PSS, with the *p* value of 0.007, 0.032, and 0.008 respectively. There was no significant difference in vertical stiffness between fractures fixed with AS and the intact model or other fixed models.

**Fig. 4:**
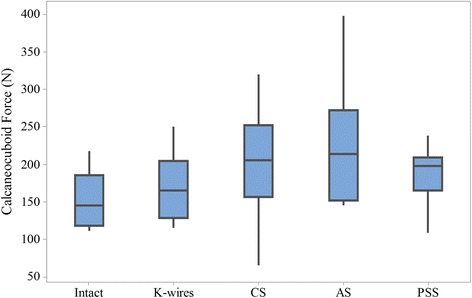
Box and whisker plots for the calcaneocuboid force of five conditions. The box indicates the interquartile range. *CS* cannulated screws, *AS* absorbable screws, *PSS* plate-screw system

Anterior–posterior stability was measured by the calcaneocuboid force. A comparison of the AP stability among all models is shown in Fig. 4. The intact model showed the lowest force of 153 (120–218; SD 39) N, while the fractures fixed with AS showed the greatest force of 242 (146–398; SD 84) N. The significance was only detected between these two models (p value 0.02).

## Discussion

A variety of techniques have been proposed for the fixation of calcaneal fractures, but the optimal fixation technique has not yet been devised, partly due to a lack of biomechanical evidence. Few reports have been published on the biomechanical performance of different implants for the fixation of intra-articular calcaneal fractures. The results presented here are of value not only for future research, but serve as an objective measure for surgeons to justify the choice of one implant over another.

Percutaneous screws fixation has been shown to disrupt less soft tissue than plating, but provide favorable clinical outcomes [6, 17–19]. There are a variety of accepted approaches for placing screws within the fractured calcaneus, but it remains unclear which configuration provides greater biomechanical strength and stiffness. It has been suggested that screws inserted perpendicular to the fracture plane could provide superior inter-fragmental fixation [14]. In our study, four cruciate screws were inserted perpendicularly to the fracture plane to attain a more stable fixation.

K-wires are commonly used for temporary intra-operative fixation, to aid in intra-operative exposure or as definitive fixation in specific patient populations. K-wires have successfully been used for the fixation of calcaneal fractures [9, 20]. Pelliccioni et al. [21] made a systematic review on the surgical treatment of intra-articular Sanders’ type II and III calcaneal fractures and found percutaneous fixation using K-wires presented the best results. However, the clinical evidence is insufficient to assert the biomechanical superiority of this treatment in comparison with other surgical techniques.

The stance phase of gait was simulated in this study to compare the stability of four implants for securing a Sander’s type III calcaneal fracture. The results showed that none of the samples failed during loading up to 700 N. This indicated that all four internal constructs potentially allow active ankle mobilization and early rehabilitation after operation without re-displacement of the fractures.

There was no significant difference in vertical stability among the models fixed with K-wires, CS or PSS, but they were weaker than the intact calcaneus. This suggested that calcaneal fractures after fixation should avoid weight bearing activities during the early post-operative stages. However, the stiffness of fractures fixed by AS was similar to that of the intact model. One possible explanation is that the calcaneal models used this study were with varying levels of bone quality. Absorbable implants are stiffer than cancellous bone [22, 23], and any bone displacements in the calcaneal models depend on the bone quality. Under the same load, the calcaneal models with good bone quality moved less than those of osteoporosis, which made the overall results comparable with the intact calcaneus. This suggested that when surgeons choose AS for calcaneal fractures, they should consider not only the fracture types but the bone quality.

Under axial compressive loading, the calcaneal fragments could move along both vertical and AP directions [24]. The AP displacement or the force at the calcaneocuboid joint could be used as an index for the assessment of stability. In previous studies, the AP stability of the calcaneus has less been mentioned. In this study, the calcaneocuboid force was measured and showed a satisfactory correlation with the vertical stability on both intact and fracture models. The intact calcaneus showed superior stability than fracture models with regard to higher stiffness and lower calcaneocuboid force. This was consistent with some biomechanical studies which compare the stability of intact and repaired bone models [25, 26]. In addition, the biomechanical stability of K-wires, CS and PSS was similar in both vertical and AP direction.

The fixation with AS was significantly less stable than the intact model. This can be explained by the weak stiffness of the AS and the small thread depth. To gain enough strength, AS are typically designed with a large diameter and small thread depth. Therefore, AS with greater strength and pull-out resistance should be encouraged to satisfy clinical needs.

Although the K-wires, CS and PSS showed similar stability in both vertical and AP stability, it should be noted that the calcaneal fractures tested were simulated fractures and without bone defects. In the clinical setting, the size and number of intra-articular fragments vary greatly among patients, and the longitudinal fracture lines of the calcaneus are sometimes much longer than in the current models. For comminuted calcaneal fractures or those with osteoporosis, PSS fixation would be the first recommendation for its framed structure and multi-point fixation. With regards to K-wires or CS, calcaneal fractures with two or three segments were the main indications and the patients deemed unsuitable for open operative treatment. The use of AS was determined by both fracture type and bone quality.

There were also some limitations to this study. Firstly, the cadaver samples used showed a large variation in bone quality, because cadaveric specimens utilized in the study were with a great range of death age. Although the use of synthetic bone could avoid the problem of osteoporosis, we believe that cadaveric calcaneus better represented the in vivo state, including compression of the subtalar joint and movement of calcaneocuboid joint. Secondly, the same calcaneus model was consecutively tested under intact and fracture conditions due to the limitation of specimen numbers. However, this procedure was common in biomechanical studies [16, 25, 27], and the simulated physiological loading would not bring much damage to calcaneus structures. Thirdly, the surrounding soft tissues were not considered in this study because the musculature was not active in the cadaveric samples. Soft tissues such as muscles, tendons and ligaments do provide additional support for the skeletal system. This might have an influence on experimental results and should be taken into account in future studies.

## Conclusions

This biomechanical study investigated the fixation strength of four different implants for treating Sanders’ types III calcaneal fractures. The global stiffness was similar when the calcaneal fractures were fixed by K-wires, CS and PSS. The stability of the fixation with AS differed along the vertical and AP directions, and was greatly influenced by the bone quality. AS for fracture fixation should be designed with greater strength and pull-out resistance.

## Abbreviations

ANOVA: analysis of variance
AP: anterior–posterior
AS: absorbable screws
CS: cannulated screws
K-wire: Kirschner wire
PLLA: poly-l-lactide acid
PSS: plate-screw system
